# Orbital cellulitis secondary to giant sino‐orbital osteoma: A case report

**DOI:** 10.1002/cnr2.1296

**Published:** 2020-10-07

**Authors:** Abbas Bagheri, Mohadeseh Feizi, Reza Jafari, Mozhgan R. Kanavi, Nasim Raad

**Affiliations:** ^1^ Ocular Tissue Engineering Research Center Shahid Beheshti University of Medical Sciences Tehran Iran; ^2^ Ophthalmic Research Center Shahid Beheshti University of Medical Sciences Tehran Iran; ^3^ Department of Ophthalmology, Faculty of medicine Mazandaran University of Medical Sciences Sari Iran; ^4^ Department of Otolaryngology, Chronic Respiratory Disease Research Center, National Research Institute of Tuberculosis and Lung Diseases Shahid Beheshti University of Medical Sciences Tehran Iran

**Keywords:** endoscopic surgery, ethmoidal osteoma, giant osteoma, orbital cellulitis, Sino‐orbital osteoma

## Abstract

**Background:**

Although osteoma is a common benign tumor of the paranasal sinuses, its orbital extension is not common. Secondary orbital cellulitis has rarely been reported in association with sino‐orbital osteoma.

**Case:**

A 30‐year‐old woman presented with left side proptosis, orbital pain and inflammation. Orbital CT scan showed a well‐defined giant osteoma in the superonasal part of the left orbit originating from the left ethmoidal sinus associated with opacity of the ipsilateral ethmoidal sinus and infiltration of orbital soft tissue. After treatment by systemic antibiotics, osteoma was resected with combined external and endoscopic surgery and the patient recovered uneventfully.

**Conclusion:**

Sino‐orbital osteoma may manifest primarily as orbital cellulitis and needs early surgical intervention.

## 1 INTRODUCTION

Osteoma, as the most common benign tumor of the paranasal sinuses,^1^ is seen in 3% of the CT scan of sinuses.^2^ Most of the sinus osteomas are asymptomatic and only 5% become symptomatic.^2^ Giant osteomas are defined as those larger than 3 cm or heavier than 110 g.^3^ These tumors may rarely extend from the sinus territory to intracranial or intraorbital spaces.^3^ The latter, based on the site and extension of the orbital involvement, may produce proptosis, diplopia, visual complaints and epiphora.^1^, ^2^, ^3^ Orbital emphysema and cellulitis have been rarely reported after extension of osteoma to the orbit.^2^, ^4^, ^5^, ^6^

Herein we report a case of orbital cellulitis secondary to orbital extension of a giant ethmoidal osteoma, which was treated with a combination of endoscopic and external approach in addition to systemic antibiotics.

## 2 CASE REPORT

A 30‐year‐old woman was referred to our clinic because of abrupt onset of proptosis, pain, blurred vision, eyelid edema and conjunctival injection of the left eye. She also complained from headache, general weakness and fever. She had a history of congenital strabismus in the left eye but no history of head trauma.

**FIGURE 1:**
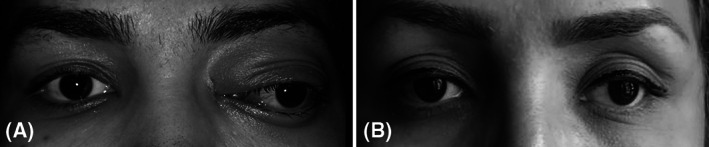
Clinical photographs of the patient. Note the presence of left‐sided extra axial proptosis, globe displacement and eyelids and conjunctival inflammation at the patient's presentation A; improvement of proptosis and inflammation at the last follow up B. Note the presence of left exotropia that remained unchanged after surgery

On general examinations, her temperature was 38.5°C. Her corrected visual acuity was 10/10 and 7/10 in the right and left eyes, respectively. She had a 5‐mm non axial proptosis and a temporal and downward displacement of the left‐sided globe. In addition to conjunctival injection and chemosis, the eyelids were edematous and inflamed in the left eye. Ocular movements of the left eye were limited in the upward and inward directions. Other ocular examinations including anterior and posterior segments were unremarkable. The afferent pupillary defect was negative (Figure 1A).

**FIGURE 2:**
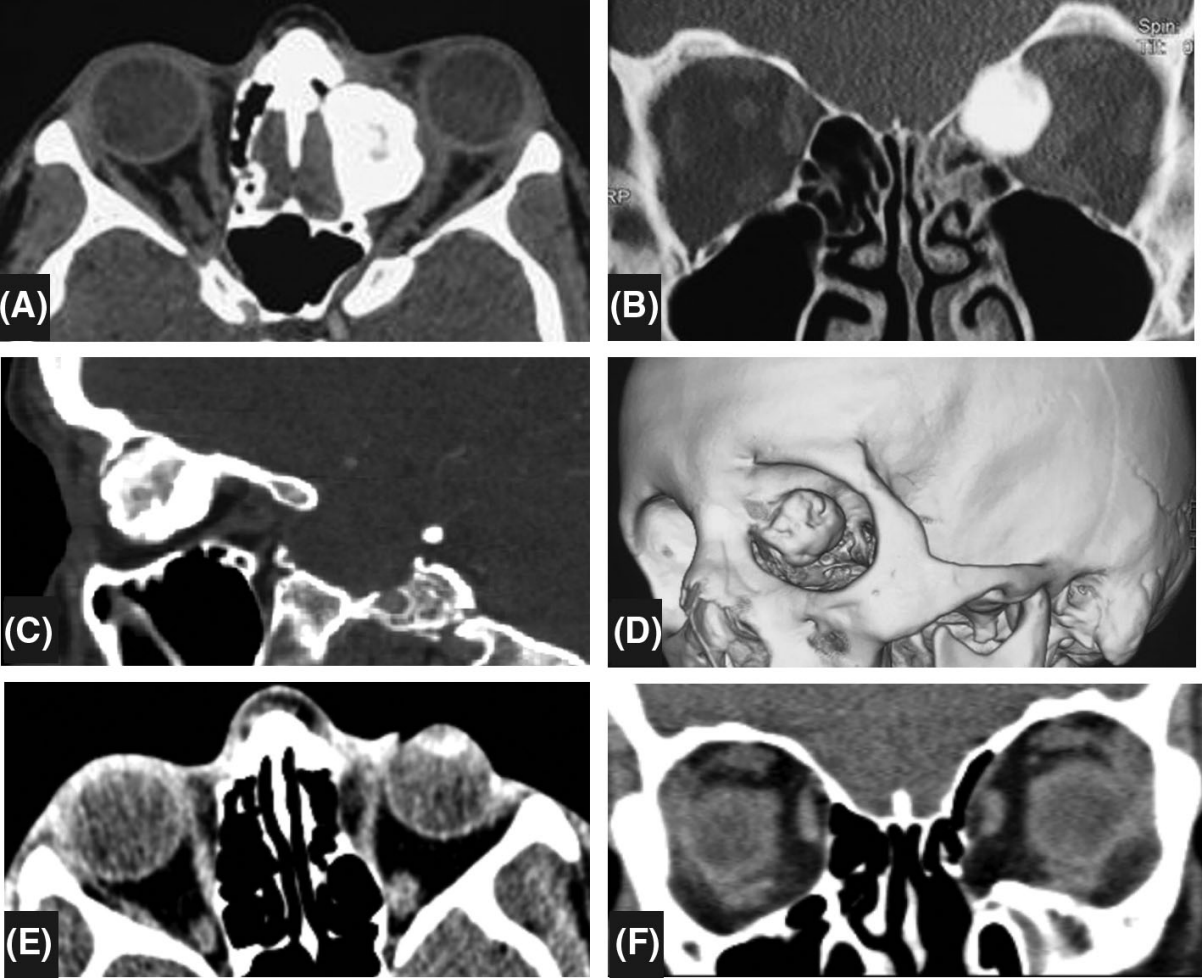
Orbital CT scan of the patient. At presentation, a superonasal giant osteoma is seen at the junction of the left frontal bone and the ethmoidal sinus, extending to the extraconal space of the left orbit. Frontal sinuses are not pneumatized and left ethmoidal sinus is opaque and partially obliterated. Compressive effect of the mass is seen on the optic nerve and orbital soft tissues. A, axial view; B, coronal view; C, sagittal view; D, three dimensional oblique view. At final follow up, the orbital walls are intact excepting the lamina papyracea and the soft tissues have returned to normal position E and F

Orbital CT scan showed a dense, heterogeneous, well‐defined and lobulated mass in the superonasal part of the left orbital extraconal space at the common border of the frontal bone and ethmoidal sinuses. As an anatomic variation, frontal sinuses were not pneumatized and left ethmoidal sinus was partially involved by the tumor. Tumor had displaced the globe to the inferotemporal space and exerted a pressure effect on the left medial rectus and optic nerve. Other findings were opacification of the left ethmoidal sinus and infiltration of the left orbital soft tissues (Figure 2A‐D).

The patient was admitted with the diagnosis of orbital cellulitis and underwent medical treatment with intravenous antibiotics including Ceftriaxone (Afachemi, Tehran, Iran) and Vancomycin (Afachemi) 1 g every 12 hours each.

**FIGURE 3:**
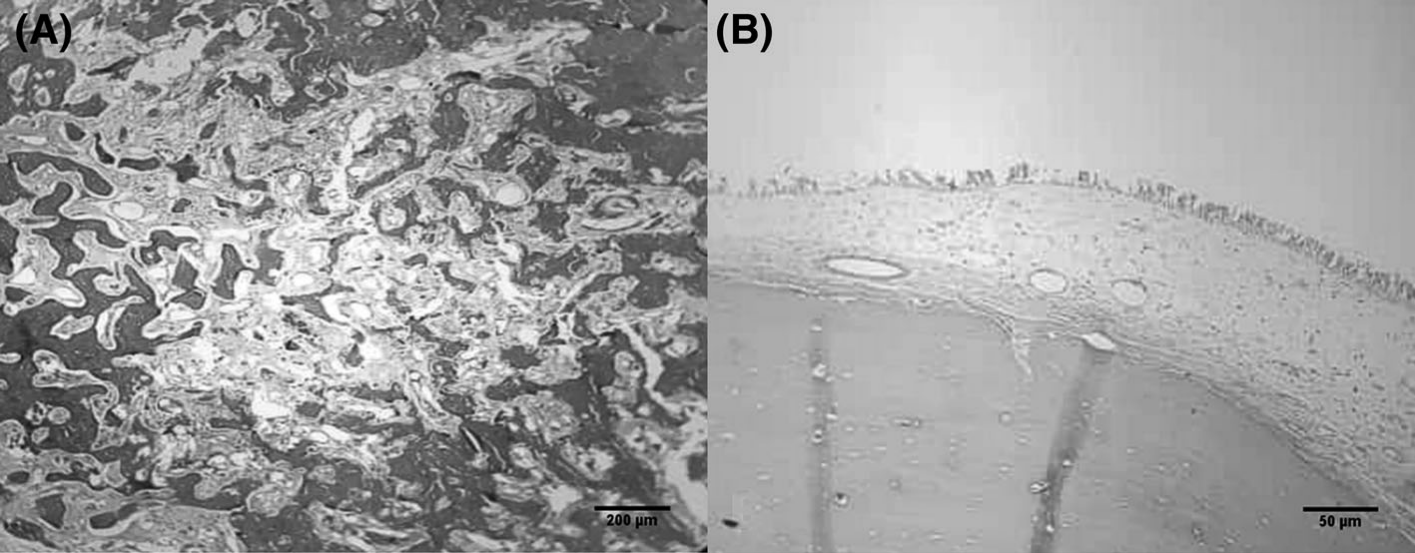
Illustrated microphotographs of the tumor showing dense mature predominately lamellar bone with peripherally located osteoblasts and inter lamellar fibro‐vascular tissue. A and B, hematoxylin and eosin

After a 3‐day systemic treatment and partial resolution of the orbital inflammation, at first the patient underwent an endoscopic approach for tumor excision under guidance of a navigation system by a team consisted of otolaryngology and oculoplastic surgeons. After left ethmoidectomy, the ethmoidal part of the tumor was released from its connections in the sinus via the transnasal endoscopic approach. Because of the high density and large size of the tumor, the en‐block excision was only possible when the external approach via the modified Lynch skin incision was added to the endoscopic method. The excised tumor was a polypoid dense white mass measuring 3.5 × 2 × 1 cm. Histopathological examinations disclosed a dense lamellar mature bone tissue with numerous peripheral osteoblasts and interosseous fibrovascular tissues, consistent with a cancellous type osteoma (Figure 3).

There was no evidence of cerebrospinal fluid rhinorrhea post‐operatively. In addition to improvement in ocular movements, the proptosis and globe displacement were completely resolved after surgery but the congenital exotropia remained unchanged. Skin incision healed beautifully and no significant scar was seen (Figure 1B).

The patient was also evaluated for Gardner syndrome by a gastroenterologist and her gastrointestinal endoscopic examinations were unremarkable. In follow up CT scan 2 years after the surgery, there was no evidence of tumor recurrence and the left orbital bony walls were intact excepting the lamina papyracea (Figure 2E, F). The patient's condition was stable up to her last (3‐year) follow up.

## 3 DISCUSSION

In this article we reported a rare case of orbital cellulitis secondary to a giant sino‐orbital osteoma and its successful management.

Osteoma is a slow‐growing mesenchymal tumor which often involves periorbital sinuses in the craniofacial area.^1^, ^2^ It is more common in the male gender and in the fronto‐ethmoidal sinuses.^1^, ^2^, ^7^

Most of the sinus osteomas are asymptomatic and can be an accidental finding of craniofacial imaging.^2^ Osteoma of paranasal sinuses rarely invade the adjacent anatomic spaces including cranium and orbit, and usually present clinically after intracranial or intraorbital extension. The reported incidence of orbital extension was 1%‐5%.^2^, ^3^

Signs and symptoms of orbital extension of sinus osteoma can be divided into two categories. The first category is the result of pressure effect of the tumor including proptosis, orbital pain, diplopia, decrease in visual acuity, palpable mass and epiphora. The second category includes clinical findings secondary to sinusitis which may be the result of obstruction or damage to the involved sinus and presenting as orbital emphysema, orbital cellulitis and subperiosteal abscess formation adjacent to the involved sinus.^2^, ^3^, ^4^, ^5^, ^6^, ^8^ The orbital extension of the infection in some reports was suggested that occurred via the bony erosion of the tumor and involvement of the orbital periosteum.^5^, ^9^ On the other hand, an intact periosteum has been shown in cases of orbital invasion of infection, suggesting that the sinus contents may gain access to the orbit not only via the emissary vessels and nerves but also through the paper like thin medial wall of the orbit.^1^, ^2^, ^3^ It was demonstrated that giant osteomas, as reported in our case, have more probability to induce sinusitis and signs of orbital invasion.^3^

Trauma, infection and developmental anomalies are suggested as the etiologic background of osteoma. In rare cases it may be seen in a genetic background such as Gardner syndrome that was rolled out in our case.^1^, ^2^, ^6^, ^10^, ^11^, ^12^

For symptomatic cases of sino‐orbital osteoma specially giant osteomas, as reported in our patient, different surgical approaches are recommended from a pure external approach to a complete endoscopic technique.^1^, ^2^, ^13^, ^14^, ^15^, ^16^, ^17^ In the recent medical literatures, difficult cases are treated by endoscopic approaches and the indications for the external approach is limited to giant dense osteomas that cannot be excised by pure endoscopic techniques.^13^, ^14^, ^15^, ^16^, ^17^ Although pure endoscopic approach, due to the less morbidities and time of hospitalization, is the preferred method for excision of the sinus osteomas, in cases with large and dense osteomas, to avoid damage to the orbital soft tissues and lengthening the operation time, the surgeon must be flexible to change the approach to the external one.^13^, ^14^, ^15^, ^16^, ^17^ A combination of external and endoscopic approaches was implemented in our patient.

In cases with obstructed sinus openings and development of sinusitis, mucocele or cellulitis in the adjacent tissues, repair of the sinus drainage pathway and returning the sinus aeration, similar to what performed in our case, are mandatory.^2^, ^5^, ^6^

Although partial removal of the sino‐orbital osteomas was reported to be successful in many cases but tumor recurrence was reported in a few cases.^2^, ^18^ With the total removal of the tumor in our case, no recurrence was observed up to a 3‐year follow up.

In conclusion orbital cellulitis is a rare complication of sino‐orbital giant osteomas, which needs urgent intervention to not only treat the infection but also to remove the tumor and establish the sinus drainage.

## ETHICS STATEMENT

The study protocol was approved by the scientific and ethics committee of the Ophthalmic Research Center at Shahid Beheshti University of Medical Sciences and adhered to the principles outlined in the Declaration of Helsinki. Written informed consent was obtained from patient to use her medical data and face photographs.

## CONFLICT OF INTEREST

The authors have no financial interest in the subject of this article.

## AUTHOR CONTRIBUTIONS

Abbas Bagheri: Conceptualization; data curation; investigation; project administration; supervision; writing‐original draft; writing‐review and editing. Mohadeseh Feizi: Data curation; investigation; software; writing‐original draft. Reza Jafari: Data curation; investigation; writing‐original draft. Mozhgan Rezaei Kanavi: Data curation; investigation; methodology; supervision; writing‐review and editing. Nasim Raad: Conceptualization; data curation; methodology; validation; writing‐original draft.

## Data Availability

The data that support the findings of this study are available on request from the corresponding author. The data are not publicly available due to privacy or ethical restrictions.
